# Insulinoma detection on low-dose pancreatic CT perfusion: comparing with conventional contrast-enhanced CT and MRI

**DOI:** 10.1186/s13244-025-01943-5

**Published:** 2025-03-22

**Authors:** Shiwei Luo, Xilong Mei, Youlan Shang, Jiaqi Yao, Nuerbiya Keranmu, Shaqi He, Cheng Yu, Fei Tang, Cong Li, Wenhan Yang, Jun Liu

**Affiliations:** 1 Department of Radiology, The Second Xiangya Hospital of Central South University, Changsha, China; 2 Imaging Center, The Second Affiliated Hospital of Xinjiang Medical University, Urumuqi, China

**Keywords:** Insulinomas, Diagnostic imaging, Tomography (X-ray computed), Perfusion imaging, Magnetic resonance imaging

## Abstract

**Objectives:**

To evaluate the efficacy of low-dose pancreatic CT perfusion (pCTP) in detecting insulinomas in patients with recurrent hypoglycemia, and to compare its diagnostic performance with conventional contrast-enhanced CT (CECT) and MRI.

**Methods:**

This study retrospectively collected 53 patients with recurrent hypoglycemia (28 with insulinomas; 25 without insulinomas). PCTP image analysis was conducted by two radiologists. Quantitative perfusion parameters of insulinomas vs. tumor-free pancreatic parenchyma were analyzed. For cases where both pCTP and CECT/MRI were performed, six radiologists blinded to the patients’ diagnosis independently evaluated the pCTP and CECT/MRI to determine the presence and location of insulinoma. The diagnostic performance of insulinoma detection between pCTP and CECT/MRI was compared.

**Results:**

For patients who underwent both CECT and pCTP, the sensitivity (CECT 0.167–0.333 vs. pCTP 0.667–1.000) of tumor detection was higher for five of six radiologists on pCTP than on CECT. For patients who underwent both MRI and pCTP, four radiologists showed higher sensitivity (MRI 0.400–600 vs. pCTP 0.700–0.800) of tumor detection on pCTP than on MRI, while two radiologists showed slightly lower sensitivity (MRI 0.800, 1.000 vs. pCTP 0.700, 0.900) on pCTP. Among perfusion parameters, peak enhancement, blood flow, and mean transit time exhibited higher AUC than blood volume and time to peak.

**Conclusion:**

PCTP demonstrated superior diagnostic performance in insulinoma detection among less-experienced radiologists compared to CECT and MRI, while more-experienced radiologists achieved marginally better results with MRI. These findings suggest pCTP’s potential as a complementary imaging modality, particularly beneficial for junior radiologists in insulinoma detection.

**Critical relevance statement:**

Pancreatic CT perfusion exhibited promising diagnostic performance in insulinoma detection, particularly among junior radiologists, demonstrating the potential to complement conventional imaging modalities and serve as a valuable clinical tool for the detection and localization of insulinoma.

**Key Points:**

- Accurate preoperative identification and localization of insulinomas is important for appropriate treatment.
- Peak enhancement, blood flow, and mean transit time outperformed blood volume and time to peak in insulinoma detection.
- Pancreatic CT perfusion has the potential to complement conventional imaging modalities for insulinoma detection.

**Graphical Abstract:**

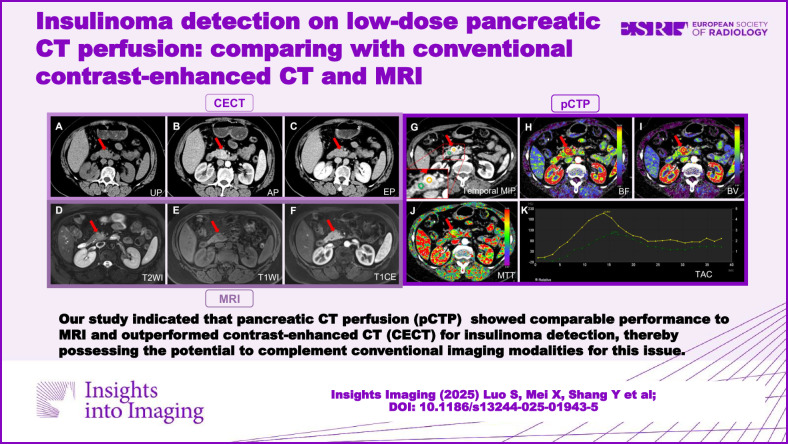

## Introduction

Hypoglycemia is a prevalent clinical manifestation, and in cases of recurrent episodes, precise identification and elimination of the underlying etiology is crucial [1, 2]. Insulinoma, a neuroendocrine tumor originating from the pancreatic tissue, constitutes one of the primary causative factors for recurrent hypoglycemia [1, 3]. It is the most prevalent subtype of functional pancreatic neuroendocrine tumors (pNETs) [4, 5]. Surgical resection remains the cornerstone of therapeutic intervention for these neoplastic lesions, underscoring the critical importance of accurate preoperative localization and characterization [5, 6]. Nevertheless, the majority of insulinomas are characterized by their diminutive size, typically presenting with diameters less than 2 cm. This inherent feature poses a significant challenge for accurate preoperative localization, thereby complicating surgical planning and intervention strategies [5]. The small size of these tumors necessitates highly sensitive and specific imaging modalities to ensure precise detection and characterization prior to surgical management.

Clinically, contrast-enhanced CT (CECT) and magnetic resonance imaging (MRI) serve as the primary preoperative imaging modalities for the localization of pNETs [3, 4, 7, 8]. Additional imaging techniques, including endoscopic ultrasonography and positron emission tomography/computed tomography (PET/CT), are frequently employed for pNETs detection [6, 9]. However, these modalities are associated with inherent limitations, including operational complexities or considerable financial costs. Typically, pNETs manifest as hypervascular lesions exhibiting a higher enhancement than pancreatic parenchyma during the arterial phase, but their small size and abundant fibrous components may result in atypical imaging features, rendering them prone to being overlooked [6]. Moreover, diagnostic challenges are exacerbated in patients with pancreatic atrophy, wherein the pancreatic parenchyma may manifest a nodular appearance and impaired vascular supply, further complicating the identification of these neoplastic lesions.

Pancreatic CT perfusion (pCTP) has emerged as a relatively novel technique that utilizes CT to evaluate the perfusion characteristics of pancreatic tissue and lesions. Previous studies have reported superior diagnostic performance of pCTP compared to conventional CECT in the evaluation of pNETs, particularly functional pNETs such as insulinomas [10–13]. However, the clinical application of pCTP has been controversial due to concerns regarding radiation exposure [11]. Recent advancements have led to the development of low-dose pCTP protocols, with emerging evidence suggesting their efficacy in the assessment of pancreatic malignancies and pNETs [14]. However, the diagnostic performance of pCTP in relation to CECT and MRI for the detection of functional pNETs has not been investigated and compared by previous studies.

In this study, we aimed to evaluate the efficacy of pCTP in the detection and localization of insulinomas in patients with clinically confirmed recurrent hypoglycemia and analyze the key quantitative perfusion parameters. Additionally, the performance of pCTP will be compared with conventional CECT and MRI to assess its potential utility in the diagnosis of insulinomas.

## Methods

### Patients

**Fig. 1:**
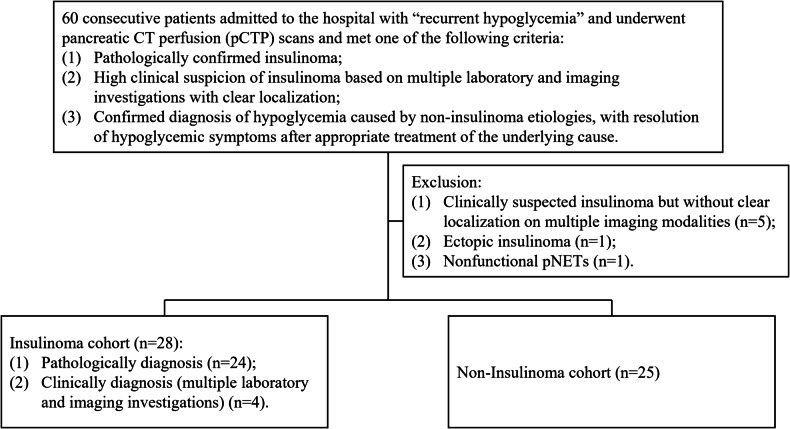
Flowchart of the study cohorts

This study was approved by our institutional review board, and the requirement for informed patient consent was waived due to the study’s retrospective nature. The study included consecutive patients who were admitted with “recurrent hypoglycemia” and undergoing pCTP scans between December 2016 and October 2023 in our institution. Inclusion criteria were as follows: (1) pathologically confirmed insulinoma; or (2) high clinical suspicion of insulinoma based on multiple laboratory investigations—including oral glucose tolerance test (OGTT), insulin secretion test, C-peptide release test—and imaging investigations with clear localization; or (3) confirmed diagnosis of hypoglycemia caused by non-insulinoma etiologies, with resolution of hypoglycemic symptoms after appropriate treatment of the underlying cause. Exclusion criteria were: (1) clinically suspected insulinoma but without clear localization on multiple imaging modalities; (2) ectopic insulinoma; (3) nonfunctional pNETs. Flowchart of the study cohorts is shown in Fig. 1.

### Image acquisition and effective radiation dose

Image acquisition for preoperative CT scans was performed using Siemens SOMATOM Force. To reduce respiratory artifacts, a belt over the abdomen was used, and patients were instructed to breathe gently during the scan acquisition. An unenhanced CT scan of the upper abdomen was performed initially to clarify the scope of the whole pancreas. The unenhanced CT scan settings: automatic tube voltage (CARE kV); automatic tube current modulation (CARE Dose4D); rotation time 0.5 s; scanning scope: the upper abdomen; FOV was determined based on the patient’s body size, typically not exceeding 350 mm; collimation: 48 × 1.2 mm; thickness: 1 mm. For the pCTP study, 40–50 mL of Iomeprol (Iomeron 400; Bracco Imaging Italia S.r.L.) was administered intravenously at a flow rate of 5 mL/s followed by 50 mL of saline solution at the same flow rate. The perfusion scan settings for most cases (excluding patients with clinically significant obesity): tube voltage 80 kV; tube current modulation 40 mAs; rotation time 0.32 s; scanning scope: the whole pancreas; collimation: 48 × 1.2 mm; thickness: 1.5 mm. The overall scan time averaged 45 s: the perfusion scan started 6 s after contrast injection, with 26 consecutive perfusion scans per 1.5 s. The detailed tube voltage and tube current of unenhanced CT and pCTP scan are shown in Table S1. The effective radiation dose (ED) was estimated from the dose length product (DLP) provided by the scanner and calculated according to the following formula:

$${{{\rm{E}}}}{{{\rm{D}}}}({{{\rm{mSv}}}})={{{\rm{DLP}}}}\,^* k$$

$${{{\rm{Conversion\; coefficient}}}}\;{k}_{{{{\rm{Abdominal\; CT}}}}}=0.015\;{{{\rm{mSv}}}}/({{{\rm{mGy}}}}\,^ * {{{\rm{cm}}}})$$

### Imaging analysis and measurement

Image analysis and measurement were processed on the professional workstation Syngo.via by two radiologists (Luo S. and Shang Y.) separately. The images were analyzed by the CT body perfusion module, motion correction was performed first, followed by segmentation (noise reducing) and vessel definition, and finally region of interest (ROI) placement and results obtaining. The ROI should be placed on the suspected pancreatic tumor location and normal pancreatic parenchyma (head, neck, body, and tail), avoiding blood vessels, necrosis, calcification, and marginal areas to minimize partial volume effect. The corresponding quantitative pCTP parameters were obtained, including blood flow (BF, the volume of blood flowing through a given volume of tissue per unit time), blood volume (BV, the total volume of blood within the vessels of a given volume of tissue), mean transit time (MTT, the average time blood takes to traverse through the tissue vasculature), peak enhancement (Peak, the maximum contrast enhancement value reached in the time-attenuation curve), and time to peak (TTP, the time from contrast arrival to peak enhancement). Pseudo-colored perfusion parametric maps and time-attenuation curves (TAC) were automatically generated. These parameter values were averaged to facilitate the subsequent calculation and analysis.

### Radiologist diagnosis

For cases where both pCTP and conventional CECT/MRI were performed, six radiologists (Yao J.—radiologist 1, Yu C.—radiologist 2, He S.—radiologist 3, Tang F.—radiologist 4, Li C.—radiologist 5, and Yang W.—radiologist 6, who had 7, 5, 3, 13, 3, and 6 years of experience in abdominal radiology, respectively), blinded to the patients’ diagnoses, independently evaluated the pCTP and CECT/MRI to determine the presence of insulinoma and record the tumor location. The evaluations of pCTP, MRI, and conventional CECT were separated by an interval of 10–14 days for the same radiologist to avoid potential bias. The details on the specific interval and order of exams per subject are shown in Table S2. Conventional CECT scans were conducted using a variety of CT scanners, including GE Revolution CT, Siemens SOMATOM Definition Flash, Siemens Sensation 64, and Siemens SOMATOM Perspective. Similarly, MRI scans were performed on different MR scanners, including Siemens Skyra, uMR 790, and Philips Achieva.

### Statistical analysis

Data analysis was conducted using SPSS software (version 26.0, SPSS Inc.). The normality of quantitative data was assessed using the Shapiro–Wilk test. The independent sample *t*-test was utilized to compare independent quantitative data with a normal distribution, while the Mann–Whitney *U* test was used for the data that did not conform to a normal distribution. The Wilcoxon signed rank test was employed for the comparison of paired samples that were not normally distributed. Chi-square test or Fisher exact test was applied to categorical variables. The performance was evaluated by accuracy (ACC), sensitivity (SEN), specificity (SPE), positive predictive value (PPV), and negative predictive value (NPV). Receiver operating characteristic (ROC) curves were constructed to evaluate the diagnostic efficacy of parameters, and the area under the curve (AUC) was calculated with 95% confidence intervals (CI). Fleiss’s Kappa coefficient was utilized to evaluate inter-rater reliability among the six radiologists, where kappa values of < 0.20 indicated poor agreement, 0.21–0.40 indicated fair agreement, 0.41–0.60 indicated moderate agreement, 0.61–0.80 indicated substantial agreement, 0.81–0.99 considered almost excellent agreement. *p*-values less than 0.05 were considered statistically significant for all statistical tests.

## Results

### Demographics

As is shown in Fig. 1, a total of 60 patients met our inclusion criteria. 5 patients were excluded due to clinical suspicion of insulinoma but lacked definitive localization on multiple imaging modalities, one patient was excluded owing to an ectopic insulinoma, and one patient was excluded due to nonfunctional pNETs. Finally, 53 patients were included (28 patients in insulinoma cohort and 25 patients in non-insulinoma cohort), and the demographics and characteristics of the study cohorts are shown in Table 1. The age distribution was 47.9 ± 13.0 years old in insulinoma cohort and 53.9 ± 16.2 in non-insulinoma cohort. There were 9 male (32%) and 19 female (68%) patients in the insulinoma cohort, and 15 male (60%) and 10 female (40%) patients in the non-insulinoma cohort. The distribution of age was not significantly different (*p* = 0.196), while the distribution of sex was significantly different (*p* = 0.042). In the insulinoma cohort, the tumor diameter distribution was 1.4 ± 0.4 cm, and the most common tumor locations were the head (36%) and tail (36%) of the pancreas. Most insulinomas were diagnosed pathologically (86%). In the non-insulinoma cohort, 6 patients had underlying diabetes mellitus, while none of the patients in the insulinoma cohort had diabetes (*p* = 0.008). Regarding the presence of hypertension, smoking, and alcohol consumption, no statistically significant differences were observed between the two cohorts.

**Table 1:** Demographics and characteristics of the study cohorts

	Insulinoma cohort (*n* = 28)	Non-insulinoma cohort (*n* = 25)	*p*-value
Age (mean ± SD, year)	47.9 ± 13.0	53.9 ± 16.2	0.196^a^
Sex (*n*/%)			0.042^b^
Male	9 (32)	15 (60)	
Female	19 (68)	10 (40)	
Tumor diameter (mean ± SD, cm)	1.4 ± 0.4	-	
Tumor location (*n*/%)			
Head	10 (36)	-	
Neck	3 (11)	-	
Body	5 (18)	-	
Tail	10 (36)	-	
Diagnosis method (*n*/%)			
Pathological	24 (86)	0 (0)	
Clinical	4 (14)	25 (100)	
Grade (*n*/%)			
G1	3 (11)	-	
G2	9 (32)	-	
G3	0 (0)	-	
Unknown	16 (57)	-	
Diabetes (*n*/%)	0 (0)	6 (24)	0.008^b^
Hypertension (*n*/%)	9 (32)	8 (32)	0.991^b^
Smoking (*n*/%)	5 (18)	9 (36)	0.135^b^
Alcoholism (*n*/%)	3 (11)	2 (8)	0.736^b^

Data are presented as mean ± SD or frequency (%). *p* < 0.05 was considered statistically significant

^a^ Independent *t*-test

^b^ Chi-square test or Fisher exact test

### Effective radiation dose

As is shown in Table S3, the median DLP of unenhanced CT (uCT), pCTP, and total (including uCT, pCTP, and topogram) are 390.2 (255.8–420.2), 109.7 (78.6–134.2), and 504.4 (363.4–571.7) mGy*cm, and the median ED are 5.9 (5.8–6.3), 1.6 (1.2–2.0), and 7.6 (5.5–8.6) mSv, respectively. An example of scan protocols and DLPs of pCTP and conventional CECT in the same patient is shown in Fig. S1; the total DLP of pCTP (507 mGy*cm) was lower than that of conventional pancreatic CECT (1207 mGy*cm).

### Quantitative PCTP parameters analysis in insulinoma cohort and non-insulinoma cohort

**Fig. 2:**
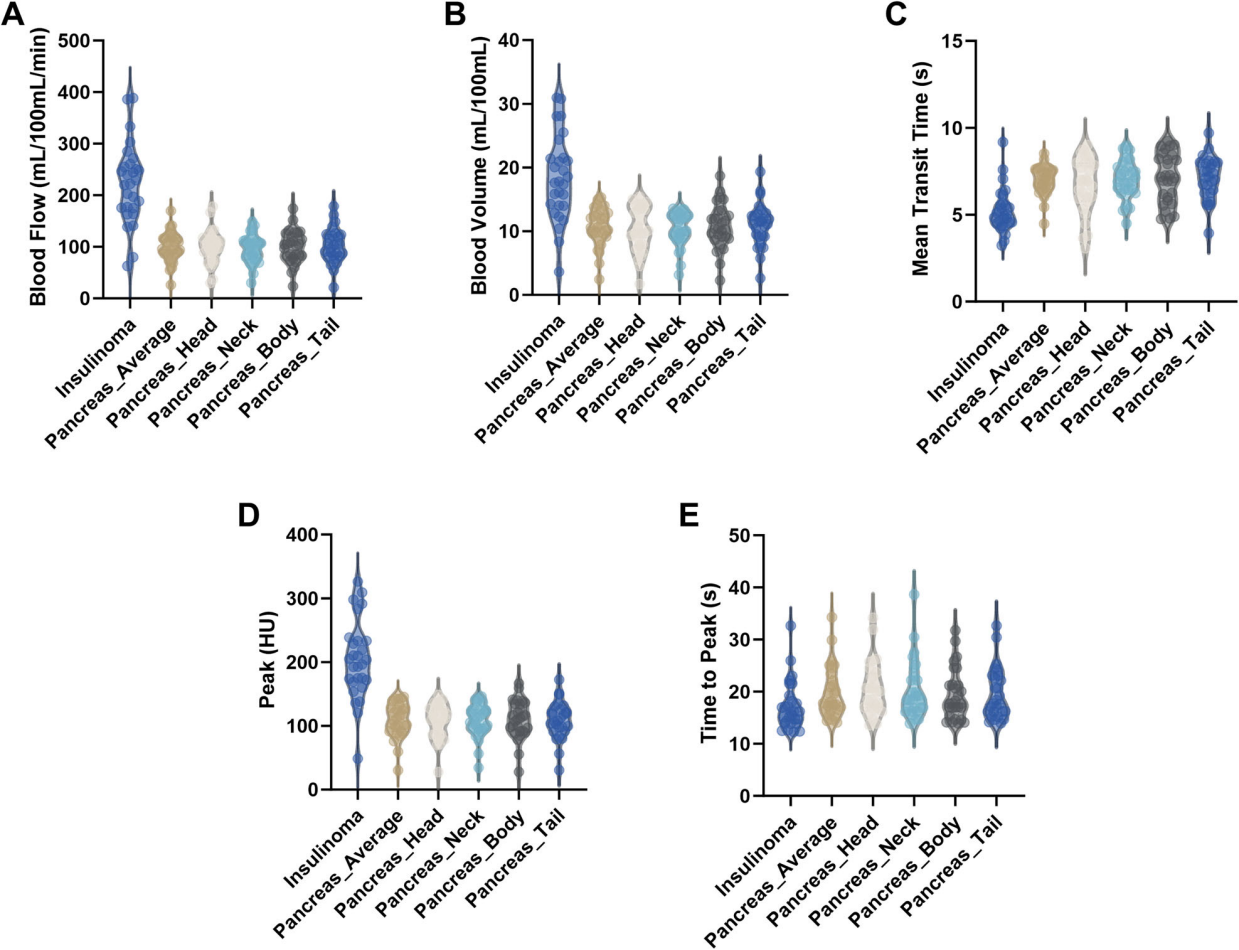
Violin plots of pCTP parameters in insulinoma cohort. **A** Blood flow (BF); **B** Blood Volume (BV); **C** Mean transit time (MTT); **D** Peak enhancement (Peak); **E** Time to peak (TTP)

The quantitative pCTP parameters of tumor and pancreatic parenchyma in insulinoma cohort are shown in Table 2 and Fig. 2. Wilcoxon signed rank test was employed as the quantitative parameters of different pancreatic region in the same patient were paired samples and did not conform a normal distribution. The BF, BV, and Peak of tumor (insulinoma) were significantly higher than that of pancreatic head, neck, body, tail, and average (Wilcoxon signed rank test, all the *p*-values were less than 0.001). On the contrary, the MTT and TTP of tumor were significantly shorter than that of pancreatic head, neck, body, tail, and average (Wilcoxon signed rank test, all the *p*-values were not higher than 0.001). Quantitative analysis revealed that the BF, BV, and Peak of the tumor were 132%, 68%, and 82% higher than that of the pancreatic parenchyma separately, while the MTT and TTP of the tumor were 29% and 9% shorter than that of the pancreatic parenchyma separately.

**Table 2:** PCTP parameters in insulinoma cohort

	BF (mL/100 mL/min)	BV (mL/100 mL)	MTT (s)	Peak (HU)	TTP (s)
Tumor	222.2 (175.6–258.0)	17.8 (14.0–21.5)	5.0 (4.5–5.9)	203.8 (170.7–233.3)	16.2 (14.2–18.7)
Head	103.7 (78.5–117.6)	10.1 (7.8–13.8)	6.9 (5.7–8.0)	111.9 (86.8–129.7)	19.9 (16.8–23.3)
Neck	101.8 (78.4–112.3)	10.1 (8.9–12.5)	7.2 (6.4–7.7)	111.6 (96.0–125.7)	18.4 (16.2–22.6)
Body	104.1 (81.0–127.0)	10.5 (9.0–13.3)	7.1 (5.9–8.3)	112.3 (91.9–135.5)	17.7 (15.0–21.3)
Tail	103.9 (82.1–124.0)	11.5 (9.2–12.8)	7.2 (6.2–8.0)	109.8 (93.3–129.5)	17.7 (15.5–23.0)
Average	95.8 (85.4–118.2)	10.6 (9.4–13.1)	7.0 (6.5–7.6)	112.2 (91.5–130.5)	17.9 (16.4–22.7)
Ratio (Tumor-average)/average × 100% (%)	132	68	−29	82	−9

Data are presented as medians, with 25th and 75th interquartile ranges (IQRs) in parentheses

Average: the average value of above pancreatic parenchyma (including head, neck, body, and tail)

*BF* blood flow, *BV* blood volume, *MTT* mean transit time, *Peak* peak enhancement, *TTP* time to peak

The pCTP parameters of pancreatic parenchyma in non-insulinoma cohort are shown in Fig. S2 and Table S4. Mann–Whitney *U* test was employed as the quantitative parameters of tumors in insulinoma cohort and different pancreatic region in non-insulinoma cohort were independent and did not conform a normal distribution. When compared to tumor (insulinoma), the BF, BV, and Peak of pancreatic head, neck, body, tail, and average were significantly lower than that of tumor (Mann–Whitney *U* test, all the *p*-values were less than 0.001). On the contrary, the MTT of pancreatic head, neck, body, tail, and average was significantly longer than that of tumor (Mann–Whitney *U* test, all of the *p*-values were less than 0.01), and the TTP of pancreatic head, neck, body, tail, and average were longer than that of tumor (Mann–Whitney *U* test, *p*-values were less than 0.01).

**Fig. 3:**
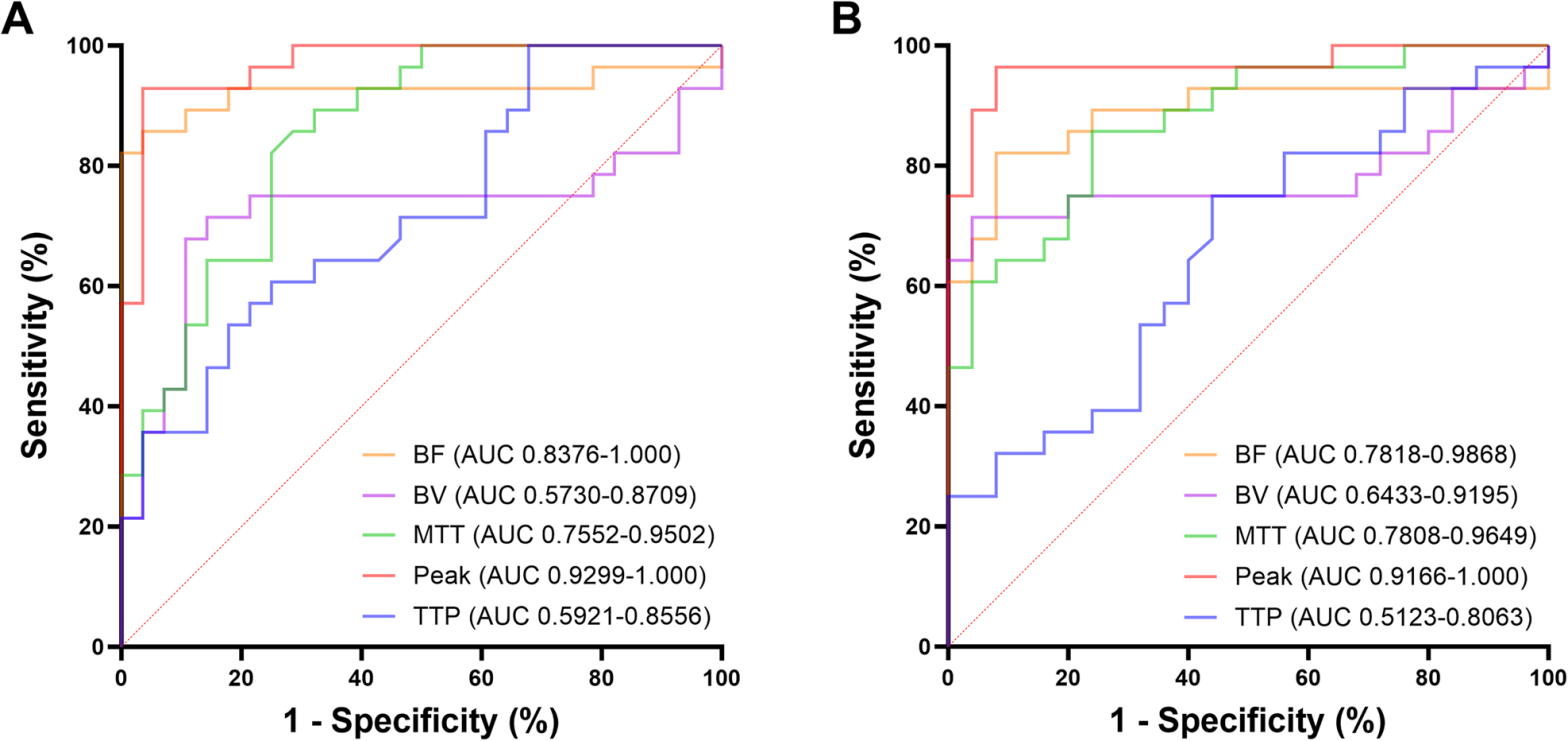
ROC curve of relative pCTP parameters in differentiating between insulinoma and pancreatic parenchyma of insulinoma cohort (**A**) and non-insulinoma cohort (**B**)

To simulate potential bias that may arise during the process of measuring quantitative perfusion parameters, for BF, BV, and Peak, we compared the minimum relative tumor value (i.e., the difference between the tumor measurement and the highest pancreatic measurement) with the maximum intrinsic pancreatic difference (i.e., the difference between the maximum and minimum measurements across different pancreatic regions) to obtain the ROC curve. Similarly, for MTT and TTP, we compared the minimum relative tumor value (i.e., the difference between the tumor measurement and the lowest pancreatic measurement) with the maximum intrinsic pancreatic difference (i.e., the difference between the minimum and maximum measurements across different pancreatic regions) to obtain the ROC curve. The ROC analysis was performed separately for insulinoma vs. pancreatic parenchyma in insulinoma cohort and insulinoma vs. parenchyma in non-insulinoma cases. As shown in Fig. 3, results showed that Peak achieved the highest AUC for tumor identification, with AUCs (95% CI) of 0.9299–1.000 and 0.9166–1.000 for insulinoma vs. pancreatic parenchyma in insulinoma cohort and insulinoma vs. parenchyma in non-insulinoma cohort, respectively. This was followed by BF, with AUCs (95% CI) of 0.8376–1.000 and 0.7818–0.9868, respectively. The AUCs (95% CI) for MTT were 0.7552–0.9502 and 0.7808–0.9649, respectively. In contrast, BV and TTP exhibited relatively inferior performance, with AUCs (95% CI) of 0.5730–0.8709 and 0.5921–0.8556, respectively, for insulinoma vs. pancreatic parenchyma in insulinoma cohort and 0.6433–0.9195 and 0.5123–0.8063, respectively, for insulinoma vs. parenchyma in non-insulinoma cohort.

### Radiologists’ diagnosis on CECT vs. pCTP and MRI vs. pCTP

**Fig. 4:**
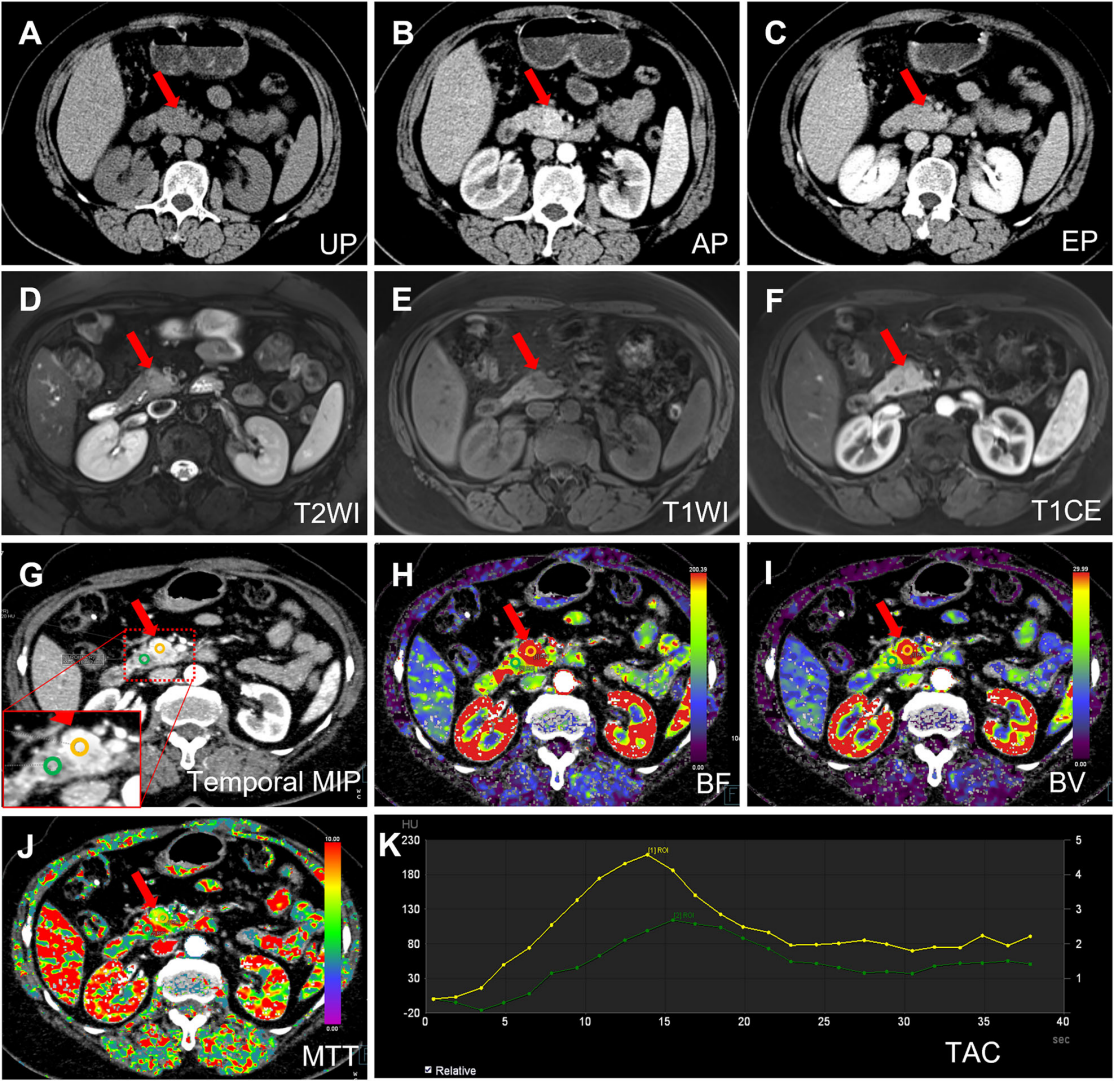
A 65-year-old female (No. 16 in Table S5) was admitted for examination after experiencing recurrent hypoglycemia. The first row shows CECT images, including unenhanced phase (UP) (**A**), arterial phase (AP) (**B**), and excretory phase (EP) (**C**). The second row displays MR images, comprising T2-weighted image (T2WI) (**D**), T1-weighted image (T1WI) (**E**), and contrast-enhanced T1-weighted arterial phase image (T1CE) (**F**). The third and fourth rows present pCTP images, including temporal maximum intensity projection (MIP) (**G**), color-coded blood flow (BF) (**H**), color-coded blood volume (**I**), mean transit time (MTT) (**J**) maps, and time-attenuation curve (TAC) (**K**). Yellow circle and curve indicate the ROI of tumor (ROI1) and green circle and curve indicate the ROI of normal pancreatic parenchyma (ROI2). Red arrows indicate the tumor

The average inter-rater agreement was fair for CECT (κ = 0.314; 95% CI 0.308, 0.320) and MRI (κ = 0.332; 95% CI 0.327, 0.336), and moderate for pCTP (κ = 0.595; 95% CI 0.591, 0.599). Six radiologists independently evaluated cases where both pCTP and CECT/MRI were performed, and the results were compared and shown in Table 3. For patients who underwent both CECT and pCTP, the sensitivity, accuracy, and NPV of tumor diagnosis were higher for five of six radiologists on pCTP images than on CECT images. The sensitivities (CECT vs. pCTP) were 0.333 vs. 1.000, 0.333 vs. 1.000, 0.333 vs. 0.667, 1.000 vs. 1.000, 0.167 vs. 0.833, and 0.167 vs. 0.833, respectively, and the accuracies (CECT vs. pCTP) were 0.429 vs. 1.000, 0.429 vs. 1.000, 0.429 vs. 0.714, 1.000 vs. 1.000, 0.286 vs. 0.857, and 0.286 vs. 0.857, respectively. The NPV (CECT vs. pCTP) were 0.200 vs. 1.000, 0.200 vs. 1.000, 0.200 vs. 0.333, 1.000 vs. 1.000, 0.167 vs. 0.500, and 0.167 vs. 0.500, respectively. In addition, for patients who underwent both MRI and pCTP, the sensitivity, accuracy and NPV of tumor detection were improved on pCTP images compared to MRI for four less-experienced radiologists, with sensitivities (MRI vs. pCTP) of 0.500 vs. 0.700, 0.400 vs. 0.700, 0.600 vs. 0.700, 0.600 vs. 0.800, accuracies (MRI vs. pCTP) of 0.538 vs. 0.769, 0.462 vs. 0.769, 0.692 vs. 0.769, 0.692 vs. 0.846, and NPV (MRI vs. pCTP) of 0.286 vs. 0.500, 0.250 vs. 0.500, 0.429 vs. 0.500, 0.429 vs. 0.600, respectively. However, for two more-experienced radiologists, the sensitivity, accuracy and NPV of tumor detection were slightly lower on pCTP images than on MRI, with sensitivities (MRI vs. pCTP) of 0.800 vs. 0.700 and 1.000 vs. 0.900, accuracies (MRI vs. pCTP) of 0.846 vs. 0.769 and 1.000 vs. 0.923, and NPV (MRI vs. pCTP) of 0.600 vs. 0.500 and 1.000 vs. 0.750, respectively. These radiologists subjectively perceived that lesion appeared more conspicuous on pCTP compared to CECT, while the major advantage of MRI was the availability of multiple sequences, facilitating the observation of lesion across various sequences. The detailed diagnostic results of each radiologist for each case are presented in Table S5. If MRI and pCTP were combined, the sensitivities for insulinoma detection were 1.000 for radiologist 1, 0.900 for radiologist 2, 0.800 for radiologist 3, and 1.000 for radiologist 4, 5, and 6. One representative case is presented in Fig. 4 (No. 16 in Table S5), only one of the six radiologists found the tumor on CECT, five radiologists found the tumor on MRI, and all radiologists found the tumor on pCTP.

**Table 3:** Diagnostic performance of insulinoma by radiologists on CECT vs. pCTP and MRI vs. pCTP

	CECT vs. pCTP (*n* = 6)					MRI vs. pCTP (*n* = 13)				
	ACC_CECT_ vs. ACC_pCTP_	SEN_CECT_ vs. SEN_pCTP_	SPE_CECT_ vs. SPE_pCTP_	PPV_CECT_ vs. PPV_pCTP_	NPV_CECT_ vs. NPV_pCTP_	ACC_MRI_ vs. ACC_pCTP_	SEN_MRI_ vs. SEN_pCTP_	SPE_MRI_ vs. SPE_pCTP_	PPV_MRI_ vs. PPV_pCTP_	NPV_MRI_ vs. NPV_pCTP_
Radiologist 1	0.429 vs. 1.000	0.333 vs. 1.000	1.000 vs. 1.000	1.000 vs. 1.000	0.200 vs. 1.000	0.846 vs. 0.769	0.800 vs. 0.700	1.000 vs. 1.000	1.000 vs. 1.000	0.600 vs. 0.500
Radiologist 2	0.429 vs. 1.000	0.333 vs. 1.000	1.000 vs. 1.000	1.000 vs. 1.000	0.200 vs. 1.000	0.538 vs. 0.769	0.500 vs. 0.700	0.667 vs. 1.000	0.833 vs. 1.000	0.286 vs. 0.500
Radiologist 3	0.429 vs. 0.714	0.333 vs. 0.667	1.000 vs. 1.000	1.000 vs. 1.000	0.200 vs. 0.333	0.462 vs. 0.769	0.400 vs. 0.700	0.667 vs. 1.000	0.800 vs. 1.000	0.250 vs. 0.500
Radiologist 4	1.000 vs. 1.000	1.000 vs. 1.000	1.000 vs. 1.000	1.000 vs. 1.000	1.000 vs. 1.000	1.000 vs. 0.923	1.000 vs. 0.900	1.000 vs. 1.000	1.000 vs. 1.000	1.000 vs. 0.750
Radiologist 5	0.286 vs. 0.857	0.167 vs. 0.833	1.000 vs. 1.000	1.000 vs. 1.000	0.167 vs. 0.500	0.692 vs. 0.769	0.600 vs. 0.700	1.000 vs. 1.000	1.000 vs. 1.000	0.429 vs. 0.500
Radiologist 6	0.286 vs. 0.857	0.167 vs. 0.833	1.000 vs. 1.000	1.000 vs. 1.000	0.167 vs. 0.500	0.692 vs. 0.846	0.600 vs. 0.800	1.000 vs. 1.000	1.000 vs. 1.000	0.429 vs. 0.600

*ACC* accuracy, *SEN* sensitivity, *SPE* specificity, *PPV* positive predictive value, *NPV* negative predictive value

## Discussion

This study revealed that, in patients with recurrent hypoglycemia, pCTP demonstrated improved insulinoma detection capabilities compared with conventional CECT. Five of six radiologists exhibited enhanced diagnostic sensitivity using pCTP images (sensitivity range: 0.667–1.000) compared to CECT images (sensitivity range: 0.167–0.333), while the other radiologist achieved equivalent diagnostic sensitivity (both 1.000) on pCTP and CECT. Both pCTP and MRI exhibited distinct advantages in insulinoma detection. Four of six radiologists achieved higher diagnostic sensitivity with pCTP (sensitivity range 0.700–0.800) compared to MRI (sensitivity range 0.400–0.600), while two of six radiologists showed marginally superior sensitivity with MRI (0.800 and 1.000, respectively) versus pCTP (0.700 and 0.900, respectively). These findings suggest that pCTP may serve as a complementary modality to MRI and CECT in insulinoma detection. Among the quantitative pCTP parameters evaluated, Peak, BF, and MTT demonstrated superior performance compared to BV and TTP. Additionally, clinical factors, such as the presence of underlying diabetes mellitus, were found to provide valuable diagnostic reference.

Insulinomas are relatively rare neoplasms, and several previous studies have employed CT perfusion or other advanced medical imaging technology for their evaluation [4, 12, 15–17]. Zhu et al [18] determined the frequency of iso-attenuating insulinomas and assessed their regional pancreatic perfusion characteristics. Results showed that the frequency of iso-attenuating tumors was 24.9%. Iso-attenuating tumor-harboring regions had lower BF compared with hyperattenuating tumor-harboring regions, and both showed higher BF compared with tumor-free neighborhood regions (all *p* < 0.01). Although the study provides valuable insights, it was limited to only two perfusion parameters (BF and BV). Moreover, the study design presumed a pre-existing diagnosis of pNETs and focused on tumor localization, rather than addressing the clinically more prevalent scenario of determining whether a patient has pNETs—the primary and crucial step. Zhu et al [19] compared the diagnostic performance of biphasic CECT, volume perfusion CT (VPCT), and 3 Tesla MRI with diffusion-weighted imaging (DWI), in patients with clinically suspected insulinomas. Results show that 3-T MRI with DWI and VPCT are significantly more accurate than CECT for insulinoma detection, with AUC of 0.715 (CECT), 0.903 (VPCT), 0.832 (MRI without DWI) and 0.955 (MRI with DWI) for reader 1, and 0.738 (CECT), 0.895 (VPCT), 0.841 (MRI without DWI), and 0.956 (MRI with DWI) for reader 2. The study offers valuable insights, and its findings demonstrate concordance with our study’s results. However, the dose of VPCT is relatively high (tube voltage 80 kVp, tube current 150mAs, ED 20.7 mSv), and quantitative perfusion parameters are not analyzed in this study. Our study addresses these limitations by enrolling patients presenting with clinical symptoms (recurrent hypoglycemia) and employing low-dose pCTP for diagnostic evaluation, and analyzes and compares quantitative perfusion parameters. Additionally, for cases where both pCTP and conventional CECT/MRI were performed, six radiologists conducted evaluations and made comparative assessments.

Quantitative perfusion parameters of tumors and different pancreatic regions were compared in our study. The results revealed that Peak, BF, and BV were higher in insulinomas, while MTT and TTP were shorter in insulinomas compared to the pancreatic parenchyma. These findings are consistent with previous studies [12, 17]. To account for potential measurement bias, we calculated the minimum relative value of the tumor (i.e., the difference between the tumor value and the highest pancreatic region value for Peak, BF, and BV, or the difference between the tumor value and the lowest pancreatic region value for MTT and TTP) and compared it with the maximum difference within the pancreas itself. Peak demonstrated the highest AUC, followed by BF and MTT, while BV and TTP performed relatively poorly. This suggests that in clinical practice, Peak, BF, and MTT may be more reliable and reproducible parameters, whereas BV and MTT exhibit comparatively lower robustness. Clinicians should consider these potential variations in parameter reliability when utilizing pCTP for decision-making. We also evaluated the efficacy of radiologists in detecting insulinoma using pCTP, CECT, and MRI. Five of six radiologists exhibited higher sensitivity, accuracy, and NPV in diagnosing insulinomas on pCTP images compared to CECT images. They reported enhanced tumor conspicuity on pCTP, indicating superior detection capabilities for relatively small insulinomas compared to conventional CECT. This may be attributed to two factors: On the one hand, insulinomas are typically small, and conventional CECT can easily overlook lesions of diminutive dimensions. On the other hand, inter-patient heterogeneity in circulatory dynamics among patients may cause the standardized protocol of conventional abdominal CECT to miss the optimal arterial enhancement phase for individual patients. In contrast, the advantage of pCTP lies in its ability to acquire multiple time points at short intervals after contrast administration, effectively capturing the maximal tumor enhancement phase and facilitating tumor detection. Four less-experienced radiologists showed higher sensitivity, accuracy, and NPV in diagnosing insulinomas on pCTP than on MRI, while two more-experienced radiologists exhibited slightly lower sensitivity, accuracy, and NPV on pCTP compared to MRI. They noted that pCTP and MRI each had distinct advantages: in pCTP, the time MIP images serve as readily interpretable indicators when combined with time-attenuation curves and quantitative parameters such as BF, BV, and MTT, these features can facilitate insulinoma detection; the strength of MRI lies in its multiple sequences, which enable enhanced lesion characterization. This suggests that pCTP and MRI could serve as complementary modalities in insulinoma detection.

There are several limitations to the study. First, this is a retrospective, single-center study with a relatively small sample size, which may have introduced selection bias and potentially limited the generalizability of the findings, requiring larger, multi-center studies to validate and extend our results across diverse patient populations and clinical settings. Second, the inclusion criteria included cases without pathological diagnosis, which may introduce potential bias in disease classification. However, it is important to note that insulinomas can be definitively diagnosed based on clinical examinations, such as biochemical tests and hormone assays. Our research primarily focused on evaluating the effectiveness of different imaging modalities in detecting insulinomas solely based on imaging findings. Third, cases where insulinomas were clinically suspected but not localized by any imaging modality were excluded, which may impact the sensitivity. This exclusion criterion, while necessary for our comparative analysis, potentially overestimates the true diagnostic performance of the imaging modalities in clinical practice. Fourth, “low-dose” pCTP is a relative and subjective concept, which refers to the lower dose of perfusion CT than the dose of conventional enhanced scanning on the same CT scanner for the same site of the same patient. In our study, pCTP and CECT were performed on different CT scanners, which may introduce variability. Fifth, the varying levels of expertise in abdominal MRI interpretation of six radiologists were not evaluated, which might influence the comparison and results, as more-experienced readers demonstrated better results with MRI. Furthermore, the interval and order of radiologists’ evaluations on CECT, MRI and pCTP could introduce additional biases. In our future research, we will take these into consideration and incorporate more radiologists with varying levels of MRI interpretation experience to enhance the scientific rigor and robustness of our findings. Additionally, we will implement strategies to minimize potential biases related to the evaluation process and imaging modality comparisons.

In summary, our study suggests that pCTP exhibits favorable diagnostic performance in insulinoma detection in patients presenting with recurrent hypoglycemia, particularly among less-experienced radiologists. It has the potential to complement conventional MRI/CECT and could serve as a valuable clinical tool for the detection and localization of insulinomas.

## Supplementary information

ELECTRONIC SUPPLEMENTARY MATERIAL

## Data Availability

The datasets generated and analyzed during the current study are not publicly available but are available from the corresponding author on reasonable request.

## Abbreviations

BF: Blood flow
BV: Blood volume
CECT: Contrast-enhanced CT
DLP: Dose length product
ED: Effective radiation dose
MTT: Mean transit time
NPV: Negative predictive value
pCTP: Pancreatic CT perfusion
pNETs: Pancreatic neuroendocrine tumors
PPV: Positive predictive value
TTP: Time to peak
uCT: Unenhanced CT
