# Supplementary material for: Insulinoma detection on low-dose pancreatic CT perfusion: comparing with conventional contrast-enhanced CT and MRI
Source: Insights Imaging. 2025 Mar 22;16:63. doi: 10.1186/s13244-025-01943-5 (PMC11929649; doi:10.1186/s13244-025-01943-5)
Supplement: Supplementary file 1 — ELECTRONIC SUPPLEMENTARY MATERIAL [file 13244_2025_1943_MOESM1_ESM.docx]

**Insulinoma detection on low-dose pancreatic CT perfusion: comparing with conventional contrast-enhanced CT and MRI**

*Supplementary Information*

**Table S1.** Tube voltage and tube current of unenhanced CT (uCT) and pCTP scan (n=53).

| Tube voltage_pCTP | |  | Tube current_pCTP | |  | Tube voltage_uCT | |  | Tube current_uCT ( |
| --- | --- | --- | --- | --- | --- | --- | --- | --- | --- |
| (kV) | Number of patients |  | (mAs) | Number of patients |  | (kV) | Number of patients |  | Mean ± SD (mAs) |
| 70 | 8 |  | 40 | 47 |  | 80 | 2 |  | 159.34 ± 37.55 |
| 80 | 44 |  | 60 | 2 |  | 90 | 48 |  |  |
| 90 | 1 |  | 70 | 1 |  | 100 | 3 |  |  |
|  |  |  | 75 | 1 |  |  |  |  |  |
|  |  |  | 80 | 2 |  |  |  |  |  |

**Table S2**. Specific interval and order of radiologists’ evaluations on CECT, MRI, and pCTP.

|  | First | Interval (days) | Second | Interval (days) | Third |
| --- | --- | --- | --- | --- | --- |
| Radiologist 1 (Yao J.) | pCTP | 14 | CECT | 14 | MRI |
| Radiologist 2 (Yu C.) | CECT | 14 | MRI | 14 | pCTP |
| Radiologist 3 (He S.) | CECT | 14 | MRI | 14 | pCTP |
| Radiologist 4 (Tang F.) | pCTP | 10 | MRI | 10 | CECT |
| Radiologist 5 (Li C.) | MRI | 10 | pCTP | 10 | CECT |
| Radiologist 6 (Yang W.) | pCTP | 10 | MRI | 10 | CECT |

**Table S3**. Dose length product (DLP) and effective radiation dose (ED) of scans.

|  | pCTP | uCT | Total |
| --- | --- | --- | --- |
| DLP (mGy*cm) | 390.2 (255.8-420.2) | 109.7 (78.6-134.2) | 504.4 (363.4-571.7) |
| ED (mSv) | 5.9 (5.8-6.3) | 1.6 (1.2-2.0) | 7.6 (5.5-8.6) |

Data are presented as medians, with 25^th^ and 75^th^ IQRs in parentheses.

DLP of total included that of unenhanced CT (uCT), pCTP, and topogram; ED of total was calculated according to DLP of total.

**Figure S1** An example of scan protocols and DLPs of pCTP and conventional CECT in the same patient (No. 16 in Table S5). (A) pCTP scan on SOMATOM Force; (B) unenhanced and contrast-enhanced pancreatic CT scan on Siemens Sensation 64.

**
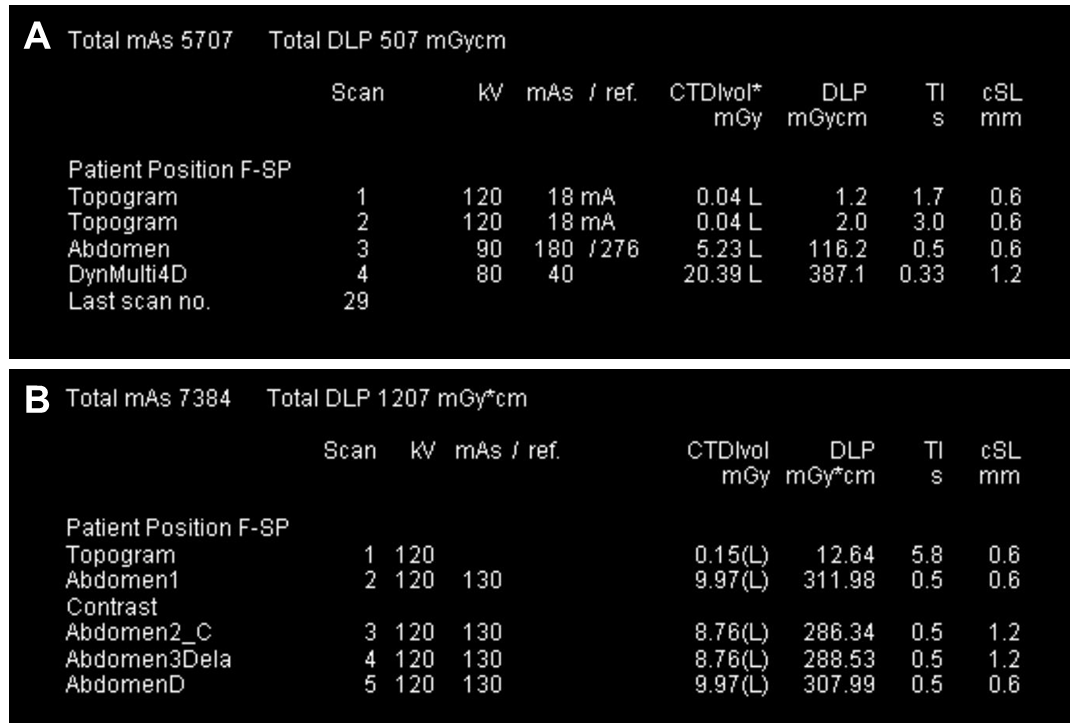
**

**Figure S2** Violin plots of pCTP parameters of pancreatic parenchyma in non-insulinoma cohort. A: Blood flow (BF); B: Blood Volume (BV); C: Mean transit time (MTT); D: Peak enhancement (Peak); E: Time to peak (TTP).


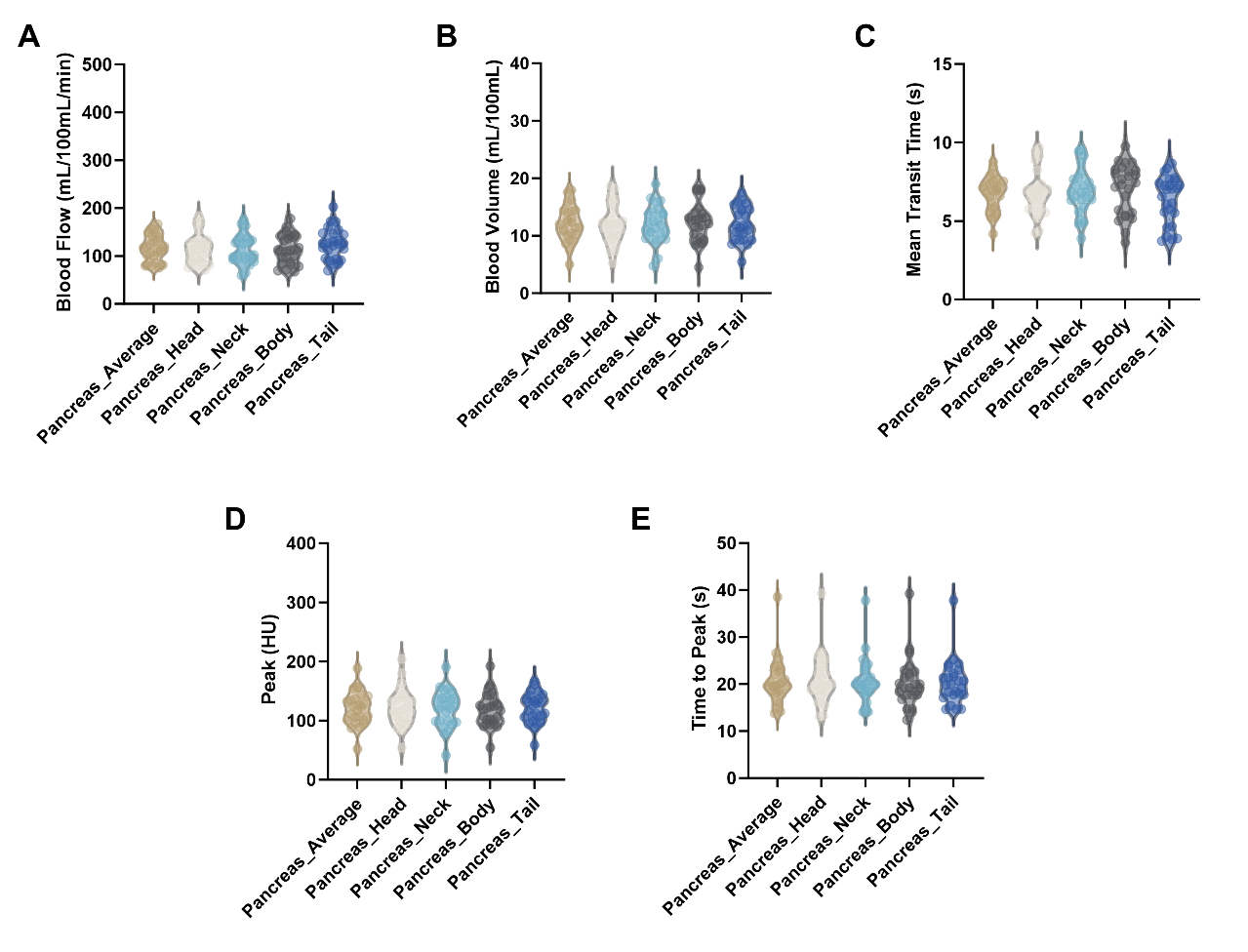


**Table S4**. PCTP parameters in non-insulinoma cohort.

|  | BF (mL/100mL/min) | BV (mL/100mL) | MTT (s) | Peak (HU) | TTP (s) |
| --- | --- | --- | --- | --- | --- |
| Head | 113.0 (90.0-131.1) | 11.8 (9.8-14.8) | 6.8 (5.8-7.2) | 117.9 (100.5-139.0) | 19.9 (18.1-23.7) |
| Neck | 102.0 (93.3-135.3) | 11.9 (9.9-14.0) | 6.9 (6.1-7.7) | 123.6 (98.1-138.0) | 20.0 (18.4-22.3) |
| Body | 112.4 (85.5-141.4) | 12.6 (9.5-14.0) | 74 (5.4-8.2) | 118.9 (98.0-136.9) | 19.2 (17.7-22.5) |
| Tail | 126.8 (93.9-147.2) | 11.8 (9.3-14.6) | 7.0 (5.1-7.5) | 125.6 (102.7-139.5) | 20.1 (17.8-22.7) |
| Average | 117.3 (93.8-140.7) | 12.1 (10.1-14.4) | 7.0 (5.9-7.5) | 123.0 (100.7-139.2) | 19.6 (18.0-22.9) |

Data are presented as medians, with 25^th^ and 75^th^ IQRs in parentheses.

BF-blood flow; BV-blood volume; MTT-mean transit time; Peak-peak enhancement; TTP-time to peak.

Average-the average value of all pancreatic parenchyma (including head, neck, body, and tail).

**Table S5**. Detailed diagnostic results of each radiologist for each case.

| No. | Radiologist 1 | | |  | Radiologist 2 | | |  | Radiologist 3 | | |  | Radiologist 4 | | |  | Radiologist 5 | | |  | Radiologist 6 | | | Truth |  |
| --- | --- | --- | --- | --- | --- | --- | --- | --- | --- | --- | --- | --- | --- | --- | --- | --- | --- | --- | --- | --- | --- | --- | --- | --- | --- |
|  | CECT | MRI | pCTP |  | CECT | MRI | pCTP |  | CECT | MRI | pCTP |  | CECT | MRI | pCTP |  | CECT | MRI | pCTP |  | CECT | MRI | pCTP |  |  |
| 1 | 1 |  | 1 |  | 1 |  | 1 |  | 1 |  | 1 |  | 1 |  | 1 |  | 1 |  | 1 |  | 1 |  | 1 | 1 |  |
| 2 |  | 0 | 0 |  |  | 0 | 0 |  |  | *1* | 0 |  |  | 0 | 0 |  |  | 0 | 0 |  |  | 0 | 0 | 0 |  |
| 3 | *0* |  | 1 |  | 1 |  | 1 |  | 1 |  | 1 |  | 1 |  | 1 |  | *0* |  | 1 |  | *0* |  | *0* | 1 |  |
| 4 | *0* | 1 | 1 |  | *0* | *0* | 1 |  | *0* | *0* | *0* |  | 1 | 1 | 1 |  | *0* | 1 | *0* |  | *0* | *0* | 1 | 1 |  |
| 5 |  | 1 | *0* |  |  | *0* | *0* |  |  | 1 | 1 |  |  | 1 | *0* |  |  | 1 | *0* |  |  | 1 | *0* | 1 |  |
| 6 |  | *0* | 1 |  |  | *0* | 1 |  |  | *0* | 1 |  |  | 1 | 1 |  |  | *0* | 1 |  |  | *0* | 1 | 1 |  |
| 7 | 0 | 0 | 0 |  | 0 | *1* | 0 |  | 0 | 0 | 0 |  | 0 | 0 | 0 |  | 0 | 0 | 0 |  | 0 | 0 | 0 | 0 |  |
| 8 |  | 1 | *0* |  |  | 1 | *0* |  |  | *0* | *0* |  |  | 1 | 1 |  |  | 1 | 1 |  |  | 1 | 1 | 1 |  |
| 9 |  | 1 | *0* |  |  | 1 | *0* |  |  | 1 | 1 |  |  | 1 | 1 |  |  | 1 | *0* |  |  | 1 | *0* | 1 |  |
| 10 |  | *0* | 1 |  |  | *0* | 1 |  |  | 1 | *0* |  |  | 1 | 1 |  |  | *0* | 1 |  |  | 1 | 1 | 1 |  |
| 11 |  | 0 | 0 |  |  | 0 | 0 |  |  | 0 | 0 |  |  | 0 | 0 |  |  | 0 | 0 |  |  | 0 | 0 | 0 |  |
| 12 |  | 1 | 1 |  |  | 1 | 1 |  |  | *0* | 1 |  |  | 1 | 1 |  |  | *0* | 1 |  |  | *0* | 1 | 1 |  |
| 13 | *0* | 1 | 1 |  | *0* | 1 | 1 |  | *0* | 1 | 1 |  | 1 | 1 | 1 |  | *0* | 1 | 1 |  | *0* | 1 | 1 | 1 |  |
| 14 | 1 |  | 1 |  | *0* |  | 1 |  | *0* |  | *0* |  | 1 |  | 1 |  | *0* |  | 1 |  | *0* |  | 1 | 1 |  |
| 15 |  | 1 | 1 |  |  | *0* | 1 |  |  | *0* | 1 |  |  | 1 | 1 |  |  | *0* | 1 |  |  | *0* | 1 | 1 |  |
| 16 | *0* | 1 | 1 |  | *0* | 1 | 1 |  | *0* | *0* | 1 |  | 1 | 1 | 1 |  | *0* | 1 | 1 |  | *0* | 1 | 1 | 1 |  |

“1” represents the presence of a tumor in the pancreas, while “0” represents the absence of a tumor in the pancreas.

Underlined and italicized text indicates an incorrect diagnosis by the radiologist.
